# A host‐free transcriptome for haustoriogenesis in *Cuscuta campestris*: Signature gene expression identifies markers of successive development stages

**DOI:** 10.1111/ppl.13628

**Published:** 2022-03-06

**Authors:** Thomas Bawin, Julien Bruckmüller, Stian Olsen, Kirsten Krause

**Affiliations:** ^1^ Department of Arctic and Marine Biology UiT The Arctic University of Norway Tromsø Norway

## Abstract

The development of the infection organ of the parasitic angiosperm genus *Cuscuta* is a dynamic process that is normally obscured from view as it happens endophytically in its host. We artificially induced haustoriogenesis in *Cuscuta campestris* by far‐red light to define specific morphologically different stages and analyze their transcriptional patterns. This information enabled us to extract sets of high‐confidence housekeeping and marker genes for the different stages, validated in a natural infection setting on a compatible host. This study provides a framework for more reproducible investigations of haustoriogenesis and the processes governing host–parasite interactions in shoot parasites, with *C. campestris* as a model species.

## 1 INTRODUCTION

Angiosperms are attacked both above and below ground by a wide variety of pathogens, including other angiosperms. Like all parasitic attacks, these plant–plant interactions can lead to considerable losses in crop yield. Within the flowering plants, the genus *Cuscuta* is one of many examples that attack other plants. With its about 200 species, it is the only parasitic member of the Convolvulaceae family and has a worldwide distribution (Costea et al., 2015; García et al., 2014). All are root‐ and leafless, and either completely lacks the ability to perform photosynthesis or show minimal photosynthetic activity of unsustainable levels (van der Kooij et al., 2000; Vurro et al., 2017). These parasites are therefore considered to be obligate stem parasites.

*Cuscuta* has a thread‐like stem that grows while rotating in a counter‐clockwise motion. As soon as it encounters a host stem, it twines around it and effectively develops lateral parasitizing organs termed haustoria on the side of the stem facing the host (Kokla & Melnyk, 2018). Early stages of haustoria promote the attachment of the parasitic shoot to the host surface. This is mediated by the production of sticky substances that allow the parasite to adhere to the host (Galloway et al., 2020; Vaughn, 2002) and coincides with a swelling of the *Cuscuta* stem and a re‐shaping of the epidermal cells into club‐shaped cells. During a successful infection on a compatible host plant, the haustorium evolves endophytic structures that penetrate the host surface, growing into the host (Johnsen et al., 2015), and enable the parasite to sequester water, inorganic salts and organic compounds, among them RNAs, proteins, hormones and metabolites (Kim & Westwood, 2015). Specialized so‐called feeding hyphae at the tip and the sides of this mature haustorium serve to connect to different cell types of the host (Shimizu & Aoki, 2019; Vaughn, 2003, 2006). Host molecules have been shown to particularly influence the formation of these feeding connections between the endophytic mature haustorium and the host (Liu et al., 2020; Narukawa et al., 2021), so that this organ with its unique cell specializations is a product of internal (i.e., *Cuscuta*‐derived) and external (i.e., host‐derived) regulating factors.

Attacks by *Cuscuta* parasites appear to go unnoticed by susceptible hosts, ostensibly because their surface chemistry and architecture are quite similar. Attempts to decipher action and reaction in the host–*Cuscuta* interaction have proven difficult, mainly since most of the infection organ is hidden from view. Early penetrating stages of the haustorium cannot be visually distinguished from feeding mature haustoria without dissecting and analyzing the infection sites. This, however, may lead to artifacts in gene expression analysis due to wound responses and degradation of RNAs in the cut tissues. In addition, the assignment of the harvested tissue samples to a development stage may differ, depending on the experimenter's judgment and morphological observation skills. The publication of the genome sequence of *Cuscuta campestris* (Vogel et al., 2018) and *Cuscuta australis* (Sun et al., 2018), the possibility to study fluorescent fusion proteins in calli (Švubová & Blehová, 2013) and the adhesive disks of the infection organs (Lachner et al., 2020), and the development of an advanced artificial host system providing control of the parasite environment throughout its life cycle (Bernal‐Galeano & Westwood, 2021) are just some of the recent milestones in parasitic plant research that helped to ascertain *Cuscuta* a role as a shoot parasitic model. In order to be able to draw conclusions across datasets from past (Ranjan et al., 2014), present (Jhu et al., 2021; this study) and future transcriptomic studies, it is highly desirable to overcome potential shortcomings in morphological sample assignment and to identify robust molecular markers that are highly specific for the different developmental stages.

Initiation and progression of haustorium formation seem to rely on several signals that are not necessarily host‐dependent and can be conveniently used to stimulate haustorium development without a living host. As Bernal‐Galeano and Westwood (2021) have recently shown, *Cuscuta* can even be grown entirely on an artificial feeding support system. The possibility to replace one highly variable factor (the host) can provide points of reference for future studies and may help to decipher how different aspects of haustorium development are controlled. The most commonly used way of host‐free haustorium induction is a combination of far‐red (FR) light and tactile stimuli (Lachner et al., 2020; Olsen et al., 2016; Tada et al., 1996), although also blue light was shown to induce haustoria (Haidar, 2003; Kaga et al., 2020). Host‐free induced haustoria are easy to monitor, and they exhibit a more uniform and predictable development compared to host‐induced haustoria. In the present study, FR induction was employed to produce non‐host‐induced haustoria of *C. campestris*, provide easy‐to‐identify hallmarks of early haustorium development and correlate gene expression patterns with characteristic morphological traits during the respective stages. Genes whose expression changed with the transition from one stage to the next were identified, and their use as haustorial stage‐defining markers was verified using independently collected on‐host samples. We present a list of robust marker genes that can be used to supplement or substitute visual stage assignment. With this study, we thus provide a framework for more reproducible investigations of haustoriogenesis and the processes governing host–parasite interactions in shoot parasites, with *C. campestris* as a model species.

## 2 MATERIALS AND METHODS

### 2.1 Plant material

Plants were maintained in a greenhouse at the Phytotron of the Arctic University of Norway, Tromsø, under 24 h daylight and approximately 21°C. *Cuscuta campestris* was originally obtained from the Botanical Garden of the University of Kiel (Germany) and propagated on *Pelargonium zonale* as compatible host. *Solanum lycopersicum* cv. M82 was grown in sphagnum peat (Veksttorv, Tjerbo, Norway) mixed at a 2:1 (v/v) ratio with perlite (Agra‐perlite, PULL Rhenen, The Netherlands). *Solanum lycopersicum* cv. M82 is tolerant to *C. campestris* attacks.

### 2.2 Host‐free haustorium induction

**FIGURE 1:**
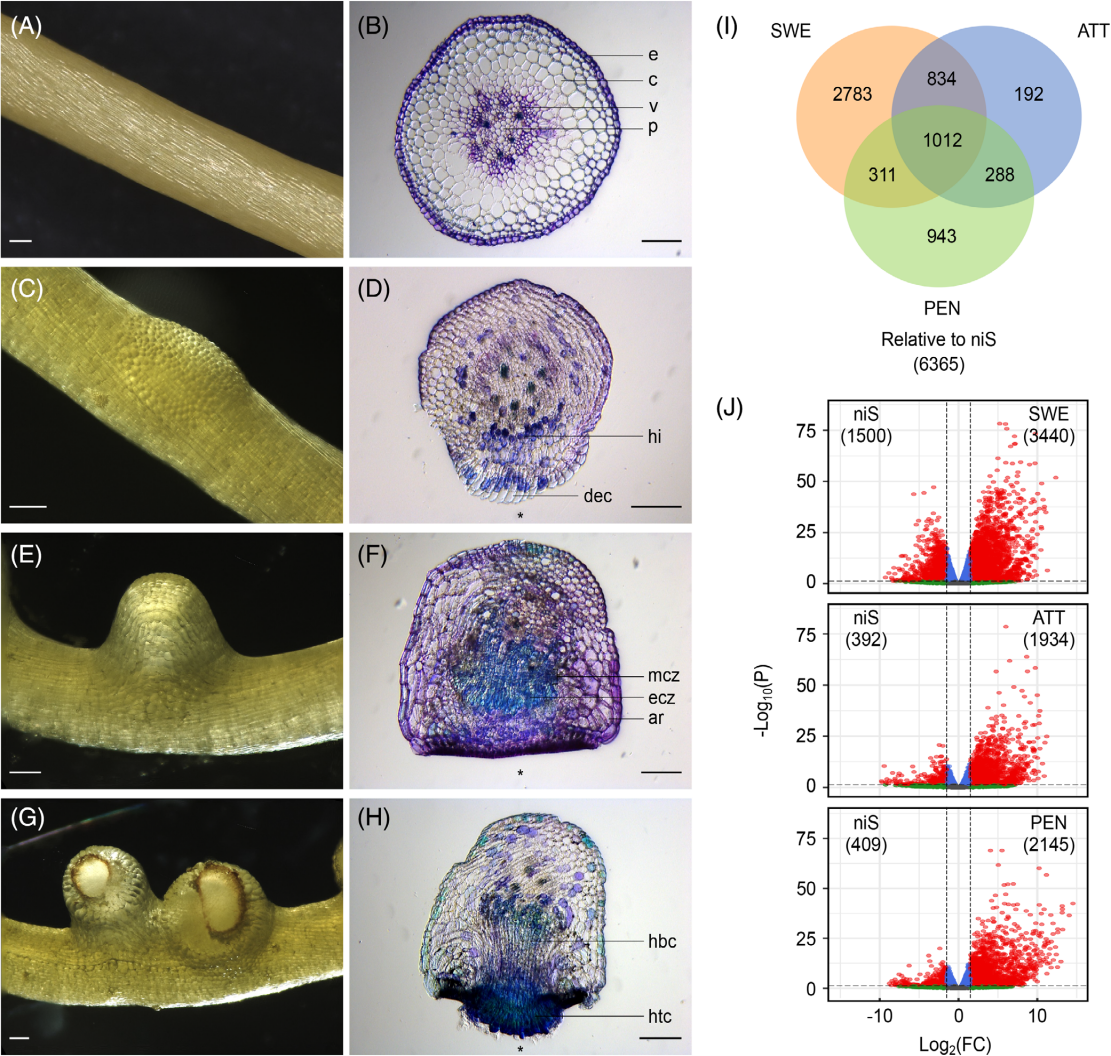
Tissue samples included in the host‐free *Cuscuta campestris* transcriptome. (A‐H) FR‐induced consecutive stages of haustorium development including non‐infecting stem (niS) (A, B), swelling stage (SWE) (C, D), attaching stage (ATT) (E, F) and penetrating stage (PEN) (G, H) were defined based on their morphological characteristics. Habitus images (A, C, E, G) and microscopy images of Toluidine Blue O‐stained cross sections (B, D, F, H) are shown for each stage. All scale bars are 150 μm. Asterisks in cross sections indicate the side facing the petri dish. (I) Representation of Venn intersections relative to niS. Numbers are overlapping differentially accumulated transcripts between pairwise comparisons of sample types. Only transcripts that have an FDR‐corrected *P*‐value ≤0.05 and a |log_2_(FC)| ≥1.5 were retained. (J) Volcano plots for differentially accumulated transcripts between infective (SWE, ATT and PEN) and non‐infective (niS) tissues. The x‐axis shows the log_2_ fold change (FC) in transcript accumulation between sample types, and the y‐axis shows the statistical significance of the differences. Horizontal and vertical dashed lines are *P*‐value ≤0.05 and |log_2_(FC)| ≥1.5 thresholds, respectively. Up‐ and downregulated transcripts that meet both criteria are highlighted in red. ar, attachment ring with large expanded cells; c, cortex; dec, digitate epidermal cells; e, epidermis; ecz, elongated cell zone of the haustorial primordium; hbc, haustorial body cells; hi, haustorial initials; htc, hyphae‐like digitate haustorial tip cells; mcz, meristem cell zone of the haustorial primordium; p, pith; v, vascular tissue

Distal portions of *C. campestris* shoots, including tips (approximately 10 cm), were harvested from individuals feeding on *P. zonale*. Host‐free induction of haustoriogenesis was carried out as described by Olsen et al. (2016) by placing them between two reversed plastic Petri dish halves (ø = 13.5 cm). Gentle pressure was applied, and halves were taped together. Shoots were placed in an upright position with their bottom into water, and irradiated with FR light (740 nm) for 2 h. They were then kept in the dark for 6 days. Shoots were finally inspected under a SteREO Lumar V12 (Zeiss) microscope equipped with an AxioCam MRc5 (Zeiss) camera. Consecutive stages of haustorium development were defined based on morphological characteristics (Figure 1A–H). Ten visually similar sites were cut from as many FR‐induced stems as needed and pooled together before frozen in liquid nitrogen. Since each FR‐treated stem contained variable numbers of haustoria in each stage, the number of stems represented in each pool differed but was at least three stems. Further, stem sections below and above areas with developing infection sites were collected using the same routine. Three biological replicates were harvested for each sample type and used for sequencing.

### 2.3 Sectioning, staining, and microscopical imaging

A Leica VT1000 E vibrating blade microtome (vibratome) (Leica) was used to produce 60 μm cross‐sections of FR‐induced haustoria. Cut sections were stained for 1 min with Toluidine Blue O (0.05% in phosphate buffered saline [PBS]) followed by destaining for 2 min in PBS. Pictures were taken using a SteREO Lumar V12 stereomicroscope (Zeiss) with an AxioCam MRc5 camera (Zeiss).

### 2.4 RNA extraction, library preparation, and sequencing

Tissue samples were homogenized using 3 mm tungsten carbide beads in a TissueLyser II (Qiagen). Total RNA extraction was performed with the RNeasy Plant Kit (Qiagen). RNA concentration and quality were measured with a Bio‐Rad Experion automated electrophoresis system using an RNA standard sensitivity kit. Library construction and mRNA sequencing were performed by Macrogen. Libraries from non‐stranded polyA mRNA (after rRNA removal) were prepared using the TruSeq RNA Sample Prep Kit v2 (Illumina), and approximately 30 M paired‐end reads (101 bp in length) per sample were produced on an Illumina NovaSeq 6000 sequencing platform.

### 2.5 Read mapping and quantification

Quality assessment of the read sequences was performed using FastQC (http://www.bioinformatics.babraham.ac.uk/projects/fastqc/). Quality trimming, adapter clipping and mapping of processed reads on the coding sequences derived from the reference genome of *C. campestris* (v. r0.31) (Vogel et al., 2018) were performed using CLC Genomics Workbench (v. 11) (Qiagen Bioinformatics). For trimming, a base‐calling error probability of 0.05, a maximum of 2 ambiguous nucleotides and a minimum length of 15 nucleotides were set as thresholds. The build‐in CLC read mapper was set to a mismatch cost of 2, an insertion/deletion cost of 3, a length fraction of 0.8 and a similarity fraction of 0.8. Raw read counts per gene were normalized to reads per kilobase million (RPKM).

### 2.6 Differential expression analysis

Differentially expressed gene transcripts were identified between sample groups using the Bioconductor software package EdgeR (v. 3.28.1) (Robinson et al., 2010). Raw read counts were used as input data, following the user's guide recommendation. Gene transcripts that had (1) a minimum of one read count in a “worthwhile” number of samples (which is defined by the software) and (2) a total of at least three counts per million across all samples were considered expressed and kept for analysis. Normalization factors were obtained using the trimmed mean of M values (TMM) method. Pairwise comparisons were based on an exact test, using the quantile‐adjusted conditional maximum likelihood (qCML) method and allowing both common and tagwise dispersion approaches. *P*‐values were adjusted using the Benjamini–Hochberg (BH) procedure (Benjamini & Hochberg, 1995). A false discovery rate (FDR) cut‐off of 0.05 and a minimum log_2_ fold‐change of 1.5 were used to retain transcripts of interest.

### 2.7 Hierarchical clustering

Two‐dimensional hierarchical clustering was performed on transcripts that were differentially regulated in at least one of any pairwise comparison of stages. Counts per million (CPM) were calculated from raw read counts, log‐transformed and converted into *z*‐score values. Clustering of both samples and transcripts was obtained by applying the complete‐linkage method to the respective Euclidean distance matrices.

### 2.8 Correlation of expression pattern to stages

Low count (noisy) transcripts were discarded by applying a minimum mean RPKM cut‐off of 2 across all samples. For each transcript, gene significance (GS) was assessed after Langfelder and Horvath (2008). Pearson correlation between transcript accumulation and simulated stage‐specific expression patterns (with 1 indicating expression in the biological samples of one stage and 0 indicating no expression in the others), was calculated. Transcripts of interest were defined as those having a minimum GS of 0.7 (on a scale of 0–1) and a significant *P*‐value (*P ≤ 0.05*).

### 2.9 Gene annotation and enrichment analysis

Assignment of *C. campestris* transcripts to MapMan4 (v.3.0) functional categories was performed using the online Mercator annotation tool (Schwacke et al., 2019). Gene set enrichment analyses were performed by applying a hypergeometric test. *P*‐values were adjusted using the BH procedure. Bins with an FDR ≤0.05 were considered significantly enriched.

### 2.10 Definition of housekeeper candidates

The following criteria were adopted: (1) expression in all samples; (2) a minimum mean log_2_(RPKM) of 5; (3) low variance, defined as lying within the 10% lower coefficient of variation (CV) and median absolute variation (MAD); (4) no significant fold change, defined as a log_2_(FC) <1.5 and an FDR >0.05 in any of the all versus all pairwise comparisons of host‐free haustorium development stages.

### 2.11 Definition of marker candidates

Differential expression and estimation of gene significance were carried out independently. By applying a reductionist approach (which ensured both high specific fold‐changes in expression and a consistent pattern among sample replicates), candidate markers of the different stages of haustorium development were defined as those that (1) were differentially expressed in all possible pairwise comparisons of a stage with the others; (2) showed a mean *z*‐score of 1 in one of the stages (indicating in our experimental settings a clear cut in expression in a sample type compared to the others); (3) satisfied the aforementioned criteria for gene significance.

### 2.12 Host plant parasitization

Distal portions of *C. campestris* shoots, including tips (approximately 10 cm), were harvested from individuals feeding on *P. zonale* and attached to *S. lycopersicum* cv. M82 stems. A continuous 16 h light, 2 h FR light and 6 h dark regime was applied. Coiled regions containing tissues of both parasite and host plants were randomly sampled. Cross‐sections were made on both sides of individual coils using razor blades. Interface regions were visually inspected under a SteREO Lumar V12 (Zeiss) microscope equipped with an AxioCam MRc5 (Zeiss) camera and assigned to one of the aforementioned stages of haustorium development. Samples containing parasites in contact with host tissues were frozen in liquid nitrogen. Cross‐sectioned non‐infective dodder stems (that were exposed to the same light regime) were further sampled in a similar way. Tissue homogenization, total RNA extraction and DNase treatment were performed as described above. RNA concentration and purity were measured with a NanoDrop ND‐1000 (Thermo Fisher Scientific) spectrophotometer.

### 2.13 Reverse transcription quantitative real‐time PCR (RT‐qPCR)

The expression of selected reference and marker candidates was measured by RT‐qPCR in both host‐free and host‐dependent samples. Gene‐specific forward and reverse primer pairs were designed using Primer3 (v. 2.4.0) (Untergasser et al., 2012). Target specificity was checked using the blastn‐short tool from the NCBI BLAST+ suite (v. 2.6.0) (Altschul et al., 1990). Both software were used as standalone to enable batch computing. The SuperScript II Reverse Transcriptase (Invitrogen) was used with anchored oligo(dT)_18_ primers to reverse transcribe DNase‐treated RNA (0.5 μg) to cDNA. Real‐time PCR was performed in 96‐well plates (Bio‐Rad/Sartorius). Thermal cycling (30 s at 95°C, followed by 40 cycles of 5 s at 95°C and 5 s at 61°C) and fluorescence detection were performed in a CFX96 Real‐Time PCR Detection System (Bio‐Rad). Individual reactions contained SsoFast EvaGreen Supermix (Bio‐Rad) (10 μL), forward and reverse gene primers (4 μL, 0.5 μM final concentration), 20× diluted cDNA (5 μL) and water (1 μL). Melt curves were generated after cycling by stepwise heating from 65 to 95°C to check amplification specificity. Negative controls obtained by adding water instead of cDNA were included. Data were analyzed using the Bio‐Rad CFX Manager software (v. 3.1). Average target abundances were calculated from technical duplicates and expressed relative to the sample with the highest expression. Relative abundances of selected housekeepers were used to normalize the expression levels between samples.

### 2.14 Evaluation of housekeeper stability

Three widely used algorithms were applied to rank the expression stability of the housekeeper candidates in both the host‐free and host‐induced systems based on their Cq values. geNorm (Vandesompele et al., 2002) was used to calculate the expression stability values M. Candidates with the lowest M‐value were the most stably expressed ones. Pairwise variations Vn/Vn + 1 were then used to determine the minimum number of reference genes required for normalization of transcript relative abundances. NormFinder (Andersen et al., 2004) was used to calculate stability values by taking both intra‐ and inter‐group variation into account, with the lowest values indicating the most appropriate candidates to be used. BestKeeper (Pfaffl et al., 2004) was used to calculate Pearson correlation coefficients, with the highest values indicating the most stable candidates. geNorm and NormFinder were implemented in a custom R‐script through the NormqPCR (v. 1.32.0) package (Perkins et al., 2012). BestKeeper was implemented by using the Microsoft Excel template provided by the authors. Rankings provided by all three methods were integrated by calculating the geometric mean for each accession.

## 3 RESULTS

### 3.1 A host‐free transcriptome for early haustorium development

When exposed to FR light in the presence of a tactile stimulus, apical portions of *Cuscuta* stems develop haustoria that in their early stages bear many of the morphological and molecular characteristics of naturally developing haustoria, as shown here for the sequenced species *C. campestris* (Figure 1A–H). These morphological signs are easily detectable under low magnification (e.g., with a stereo microscope). The first visual sign appears after one to two days with a slight bump where the otherwise flat epidermal cells appear rounded on the surface (Figure 1A,C). Cross‐sections of these sites show that in comparison to the non‐induced stems, these epidermal cells undergo a strong elongation perpendicular to the surface and haustorium initials appear in the region between the cortex and the vascular tissue (Figure 1B,D). This early infective “*swe*lling stage” (SWE), soon becomes more pronounced and culminates in a macroscopically visible structure of 1–2 mm (Figure 1E) that begins to stick to the surface it faces (Vaughn, 2002) (“*att*aching stage” [ATT]). In cross‐sections, the newly formed haustorial primordium with its meristem and elongation zones (Lee, 2007, 2008) is visible upon staining with the polychromatic stain Toluidine Blue O by its bright blue/turquoise color (indicating the presence of lignin or other polyphenolic compounds in the cell wall) (Figure 1F). The epidermal cells facing the surface are palisade‐formed and dark purple stained cells owing to their high pectin content (Vaughn, 2002). In the center of this lateral structure, the haustorium finally emerges and the adhesive surface forms a ring around it (Figure 1G,H). The emerging haustorium has digitate cells at its tip that sometimes protrude from the surface (Figure 1H) and are occasionally termed “search hyphae” (Kaga et al., 2020) although they more likely serve host penetration rather than feeding purposes. On a host, the split in the sticky adhesive ring together with the action of degrading enzymes causes a rupture in the infected tissue and the intrusion of the haustorium that will then begin to feed. In the host‐free system used here to induce haustoria, this is the final stage that can be observed and will be referred to as the “*pen*etrating stage” (PEN). FR‐exposed stem sections below and above the region where haustoria developed (*n*on‐*i*nfective *s*tems or “niS”) (with no visual changes in morphology) were used as reference sites. Twelve samples (triplicates of the aforementioned three infective and one non‐infective tissue types) were collected for sequencing, and a total of approximately 375 M raw reads for all libraries was generated. After trimming >30 M high‐quality clean reads remained in each sample. Mapping on the coding sequences derived from the gene models of the *C. campestris* genome (Vogel et al., 2018) covered 49,819 (90.1%) of the 55,311 identified transcripts (Table S1, Appendix S1). Hierarchical clustering analysis of the sequencing data confirmed the consistency between biological replicates (Figure S1).

### 3.2 Host‐free haustorium development is accompanied by major transcriptional reprogramming

The infective tissues (SWE, ATT and PEN) were compared with non‐infective stems (niS) as a background to investigate the dynamics of transcript accumulation (Figure 1I,J). This revealed that the most profound changes occurred during the transition between niS and SWE, with 3440 and 1500 transcripts (4940 in total) that were respectively up‐ and downregulated during these earliest signs of haustorium development. By contrast, only about half as many transcripts were differentially regulated in the subsequent stages: 2326 in ATT and 2554 in PEN. In both cases, the majority were upregulated (1934 and 2145 transcripts, respectively), while only a few genes were downregulated during this development (392 and 409 transcripts, respectively). While 1012 of the differentially expressed gene transcripts (DEGs) were common to the three stages, varying numbers were found to be specifically expressed in only one stage. The highest number of stage‐specific transcripts was exhibited by SWE with a total of 2783 transcripts, followed by PEN with 943 transcripts. The transitory ATT boasted only 192 transcripts that can be described as typical for this stage.

**FIGURE 2:**
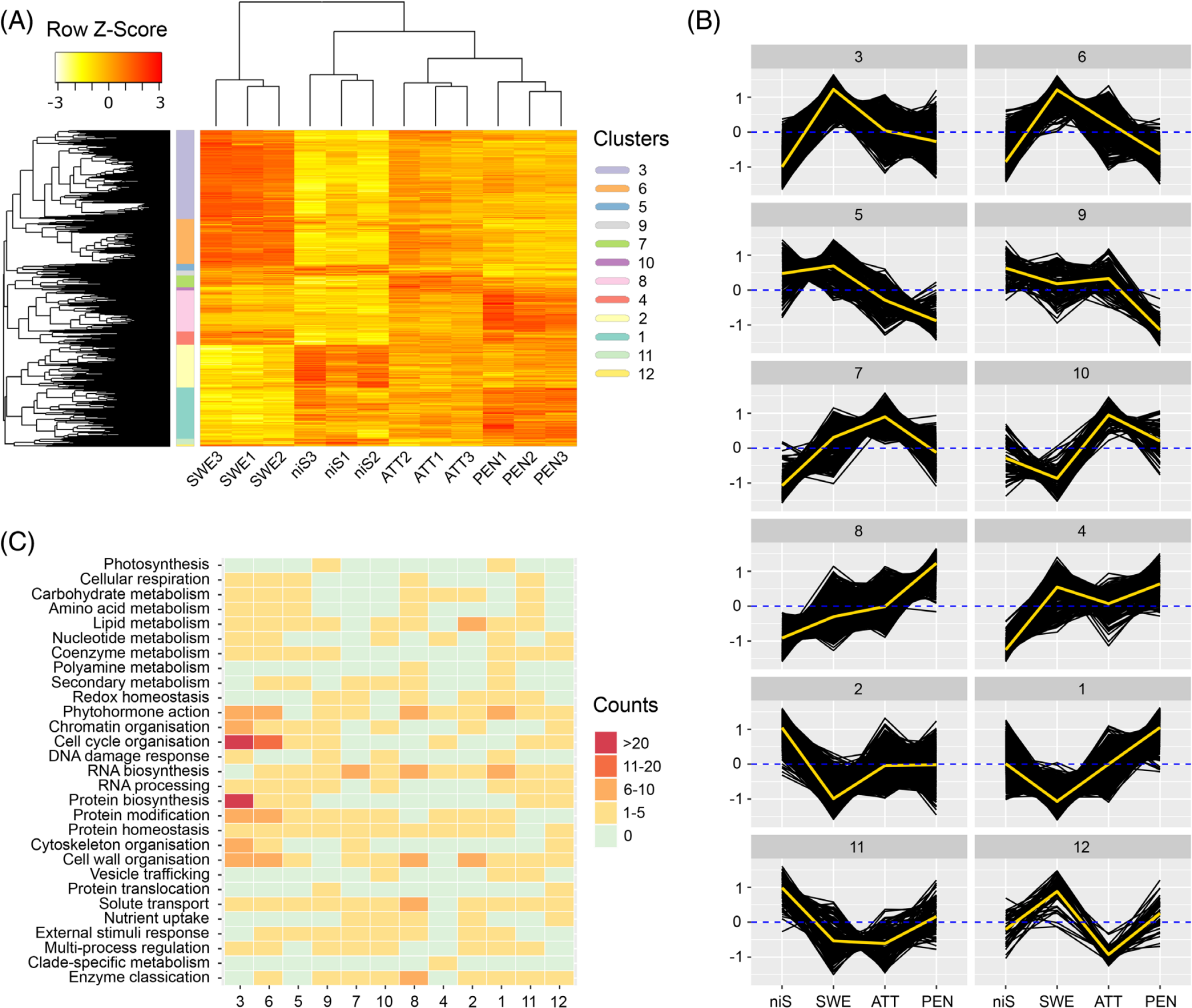
Hierarchical clustering of DEGs in host‐free haustorium development. (A) Two‐dimensional clustering of transcripts and samples identifies clusters of transcripts with similar accumulation profiles. (B) Expression in clusters as a function of haustorial development stages. Dark lines are mean transcript accumulation (transformed into *z*‐score) over three samples for each of the stages. A *z*‐score value is positive (negative) if the transcript accumulation in a sample type is larger (smaller) than the overall mean accumulation. Yellow lines summarize the mean accumulation (pattern) inside each cluster. Dashed blue lines delineate a *z*‐score value of 0. (C) Schematic representation of MapMan4 v.3.0 functional category enrichment as a function of clusters. Counts refer to the number of bins that are significantly enriched with transcripts inside each top‐level category. ATT, attaching stage; niS, non‐infective stem; PEN, penetrating stage; SWE, swelling stage

Major transcriptional dynamics during the transition from vegetative tissues to an infective structure in early haustoriogenesis were identified using a hierarchical clustering approach. A total of 7292 transcripts that were differentially regulated in at least one pairwise comparison of stages was selected. Twelve overarching clusters grouping DEGs with overall similar expression profiles were defined (Figures 2A and S2). We identified close similarities in transcriptional patterns between ATT and PEN. Interestingly, shifts in gene expression were less different between those two stages and niS than SWE, reflecting the more diverse changes in the very beginning of haustorium development. While clusters 2 and 11 gathered DEGs that showed a higher accumulation in the control stem sections without haustoria and that are repressed upon haustoriogenesis, some of the other clusters gathered genes with distinct peaks in expression at one of the haustorial development stages (Figure 2B). Clusters 3 and 6 gathered a large proportion (3076 [42.2%]) of the DEGs mainly associated with SWE. Clusters 7 and 10 grouped DEGs that were mostly upregulated in ATT. Cluster 8 was mainly associated with PEN. The transition from niS to SWE was marked by a shift toward a strong representation of MapMan4 functional bins related to phytohormones action, chromatin and cell cycle organization as well as RNA biosynthesis and processing, protein biosynthesis, modification and homeostasis, cytoskeleton and cell wall organization, and solute transport (Figure 2C). It should also be noted that almost all the metabolism‐related categories contained bins significantly enriched with DEGs. The transition from SWE to ATT showed a rather moderate number of functional categories (including RNA biosynthesis) with few representatives. Finally, the transition from ATT to PEN was associated with a distinct number of DEGs in bins related to phytohormones action, RNA biosynthesis, cell wall organization, solute transport, and enzyme classification.

### 3.3 Stage‐specific patterns of gene expression during host‐free haustorium development allow for the identification of marker genes

Gene transcripts that are expressed at a constant level during haustoriogenesis and can be used as reliable internal controls to correct for sample‐to‐sample variations in further experimental studies were first determined. Applying filtering criteria (see Section 2.10) resulted in a list of the 716 most stable transcripts from 688 gene models, which were considered as candidate housekeepers for in vivo investigation and normalization of gene expression. Among these transcripts, 5 from the top 20 that were assigned to different MapMan4 categories were selected for further investigation (Tables 1 and S2).

**TABLE 1:** Selected housekeepers for in vivo investigation and normalization of gene expression in haustorium development stages in *Cuscuta campestris*

Accession	Log_2_(RPKM) ± se	CV	MAD	Bincode	Description
Cc002986.t1	6.52 ± 0.02	0.01	0.05	15.5.30	Transcription factor (bHLH)
Cc006757.t1	6.82 ± 0.03	0.01	0.07	24.1.1.2.2	Subunit B of V‐type ATPase peripheral V1 subcomplex
Cc028378.t1	5.42 ± 0.02	0.01	0.07	13.3.4.1.2	Component SMC3/TTN7 of cohesin regulator complex
Cc028808.t1	6.16 ± 0.02	0.01	0.05	19.2.5.2.2.6	Regulatory component RPN7 of 26S proteasome
Cc036327.t1	5.57 ± 0.02	0.01	0.04	5.7.3.2.2	Multifunctional enzyme (MFP)

*Note*: Expression values are average log_2_(RPKM) ± se across all samples (four types, three biological replicates each). “CV” and “MAD” are respectively coefficient of variation (commonly used) and median absolute deviation (more robust to outliers), with a low value indicating a more stable expression. “Bincode” and “Description” refer to MapMan4 v.3.0 functional annotation.

Marker genes that can be used to assign samples to a specific stage in later analyses of parasite–host interactions were then identified. Applying filtering criteria (see Section 2) resulted in a list of 585 transcripts from 556 gene models that were considered as candidate markers for the different haustorium development stages, including 68 transcripts (67 genes) for niS, 154 (149) for SWE, 7 (7) for ATT and 356 (333) for PEN. For each stage, three marker candidates from different gene models were selected for validation (Tables 2 and S3). Due to the limited number of marker candidates for ATT, whose low accumulation level (mean RPKM <5 in most cases) in this stage made them difficult to detect by quantitative RT‐PCR techniques, the filtering criteria had to be relaxed by allowing that not all possible differential comparisons between this stage and the others were significant. This yielded Cc010463.t1 as a candidate.

**TABLE 2:** Selected markers for different stages of haustorium development in *Cuscuta campestris*

Accession	Log_2_(RPKM) ± se	Max	Min	Stage	Zscore	GS	p.GS	Bincode	Description
Cc000593.t1	10.24 ± 0.48	12.27	7.22	niS	1.17	0.70	0.011	35.1	Acidic endochitinase
Cc001625.t1	5.30 ± 0.35	7.81	3.98	niS	1.49	0.90	<0.001	27.2.4.2	Programmed cell death metacaspase‐like regulator (MCP1)
Cc019339.t1	7.66 ± 0.61	11.39	4.67	niS	1.49	0.90	<0.001	35.2	–
Cc008373.t1	1.40 ± 0.36	3.39	0.00	SWE	1.51	0.90	<0.001	13.2.1.1.6	Component ORC6 of origin recognition complex
Cc009295.t1	2.41 ± 0.37	4.40	0.67	SWE	1.44	0.88	<0.001	21.7.2	Regulatory beta‐1,3 glucanase (pdBG)
Cc015960.t1	1.33 ± 0.35	3.45	0.00	SWE	1.45	0.90	<0.001	35.1	Araport11 vacuolar import/degradation Vid27‐related protein
Cc010463.t1	1.10 ± 0.43	3.79	0.00	ATT	1.27	0.77	0.004	35.2	–
Cc019664.t1	1.19 ± 0.50	4.77	0.00	ATT	1.57	0.95	<0.001	35.2	–
Cc020138.t1	1.80 ± 0.43	4.48	0.00	ATT	1.36	0.81	0.001	15.5.7.2	Transcription factor (DREB)
Cc002183.t1	1.90 ± 0.63	5.79	0.00	PEN	1.62	0.95	<0.001	11.10.1.10.1	CLE precursor polypeptide
Cc004177.t1	2.52 ± 0.43	5.61	1.23	PEN	1.57	0.95	<0.001	50.1.1	Berberine bridge enzyme‐like
Cc008389.t1	1.53 ± 0.65	6.04	0.00	PEN	1.57	0.94	<0.001	21.4.2.1	Alpha‐class expansin

*Note*: Expression values are average log_2_(RPKM) ± se across all samples (four types, three biological replicates each). “Max” and “Min” are respectively maximum and minimum expression. “Stage” refers to the development stage toward which a marker is directed. “Zscore” refers to the average *z*‐score in the corresponding stage. “GS” and “p.GS” are gene significance and corresponding *P*‐value for that stage. “Bincode” and “Description” refer to MapMan4 v.3.0 functional annotation.

Abbreviations: ATT, attaching stage; niS, non‐infective stem; PEN, penetrating‐like stage; SWE, swelling stage.

### 3.4 Stage‐specific markers provide for the classification of natural infection sites

The expression of reference and marker candidates was investigated by quantitative real‐time PCR (RT‐qPCR) (Appendixes S2 and S3). To this end, the host‐free induction experiment was reproduced so that a new set of RNA samples from three biological replicates for each haustorial stage was obtained. Moreover, infection sites of *C. campestris* on the tomato (*S. lycopersicum*) cultivar M82 were collected, their morphology documented by microscopy after sectioning and their RNAs extracted.

The stability of the five selected reference candidates was first evaluated using three different statistical approaches: geNorm (Vandesompele et al., 2002), NormFinder (Andersen et al., 2004) and BestKeeper (Pfaffl et al., 2004). Rankings provided by all three methods were integrated by calculating the geometric mean for each accession (Table 3). In both the host‐free and host‐induced systems, only two genes were found to be sufficient for proper normalization, as indicated by the low average expression stability M (<1) and pairwise variation V2/3 (<0.2) values provided by the geNorm algorithm (Table 3, Figure S3). However, ranking integration provided contrasting results, with Cc028808.t1 and Cc006757.t1 being the most suitable pair in the host‐free system, and Cc028378.t1 and Cc002986.t1 the most suitable pair in the host‐induced one. Those two pairs were selected and further used independently in the respective systems for proper normalization of the expression of the marker candidates.

**TABLE 3:** Stability ranking of the selected reference candidates in host‐free and host‐induced systems

System	geNorm			NormFinder			BestKeeper			Total		
	Accession	Rank	M	Accession	Rank	SV	Accession	Rank	r	Accession	Rank	GM
Host‐free	Cc006757.t1	1	0.48	Cc028808.t1	1	0.32	Cc006757.t1	1	0.95	Cc028808.t1	1	1.00
	Cc028808.t1	1	0.48	Cc028378.t1	2	0.43	Cc028808.t1	1	0.95	Cc006757.t1	2	1.59
	Cc036327.t1	3	0.58	Cc036327.t1	3	0.49	Cc036327.t1	3	0.91	Cc036327.t1	3	3.00
	Cc028378.t1	4	0.78	Cc006757.t1	4	0.51	Cc028378.t1	4	0.74	Cc028378.t1	4	3.17
	Cc002986.t1	5	0.87	Cc002986.t1	5	0.52	Cc002986.t1	5	0.27	Cc002986.t1	5	5.00
Host‐induced	Cc002986.t1	1	0.29	Cc028378.t1	1	0.19	Cc028378.t1	1	0.95	Cc028378.t1	1	1.00
	Cc028378.t1	1	0.29	Cc006757.t1	2	0.27	Cc006757.t1	2	0.94	Cc002986.t1	2	1.82
	Cc006757.t1	3	0.36	Cc002986.t1	2	0.27	Cc028808.t1	3	0.88	Cc006757.t1	3	2.29
	Cc028808.t1	4	0.39	Cc028808.t1	4	0.29	Cc002986.t1	3	0.88	Cc028808.t1	4	3.63
	Cc036327.t1	5	0.51	Cc036327.t1	5	0.42	Cc036327.t1	5	0.52	Cc036327.t1	5	5.00

*Note*: “M” refers to geNorm's average expression stability value; SV refers to NormFinder's stability value; “r” refers to BestKeeper's Pearson correlation coefficient; “GM” refers to geometric mean of the rankings from the three methods.

**FIGURE 3:**
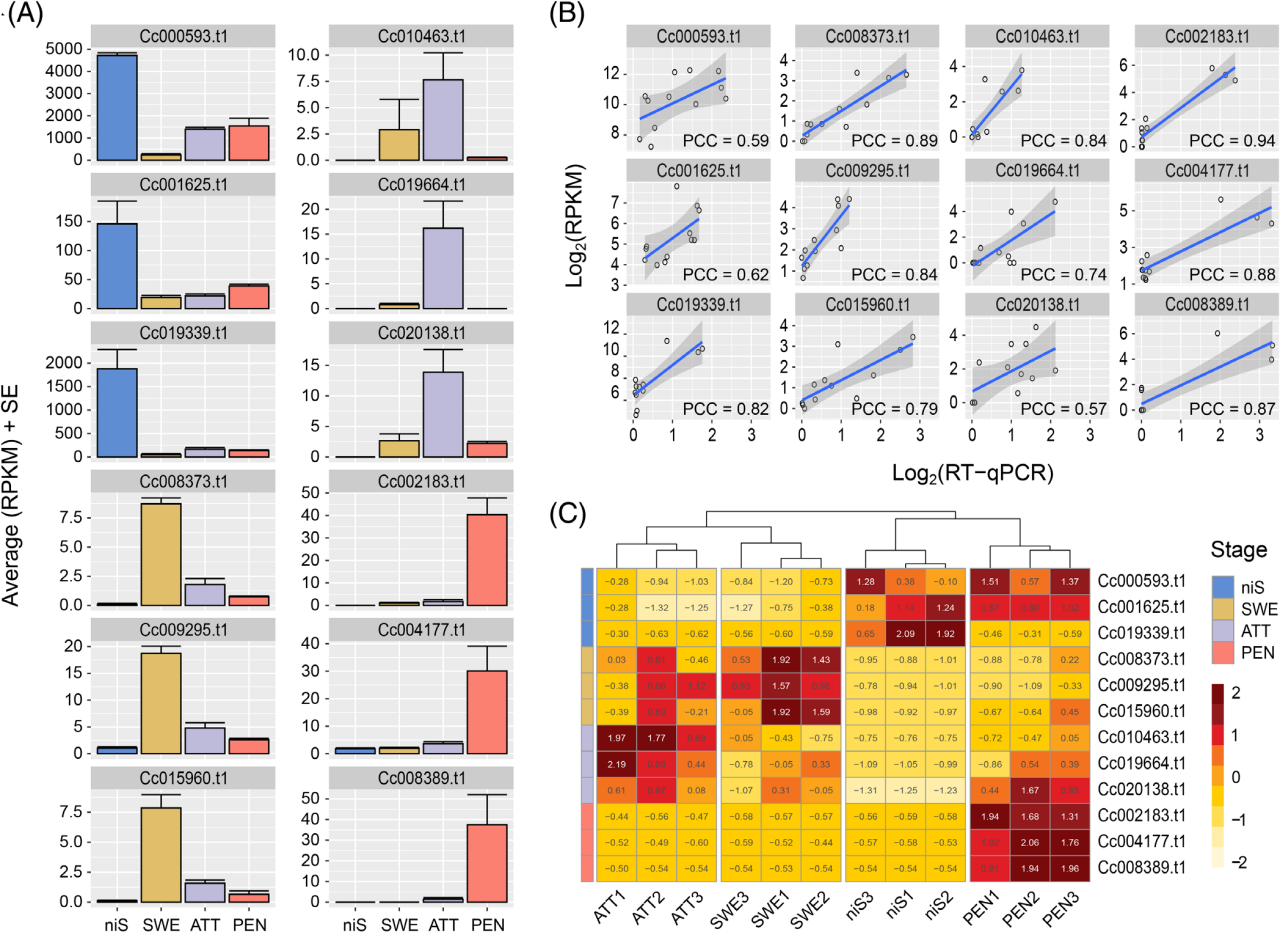
Expression profile of selected markers in host‐free haustorium development. (A) Average RPKM in sequenced samples. Error bars indicate standard error of the mean (three biological replicates). (B) RT‐qPCR validation of the sequencing data. Pearson correlation coefficients (PCC) between RPKM values in sequenced samples and transcript abundance as measured by RT‐qPCR in parallel validation samples are provided in each plot panel. RT‐qPCR data were normalized against Cc028808.t1 and Cc006757.t1. (C) Normalized RT‐qPCR transcript accumulation in host‐free parallel samples, transformed into *z*‐scores. Hierarchical clustering was based on Euclidean distance. A *z*‐score value is positive (negative) if the transcript accumulation in a sample is larger (smaller) than the mean row accumulation. ATT, attaching stage; niS, non‐infective stem; PEN, penetrating stage; SWE, swelling stage

**FIGURE 4:**
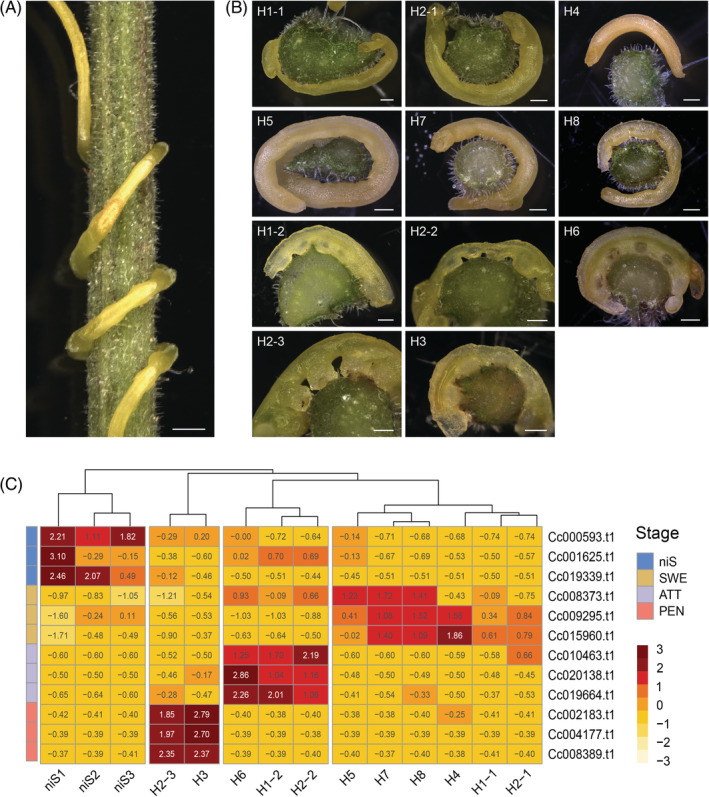
Expression profile of selected markers in host‐induced haustorium development. (A) *Cuscuta campestris* wrapped around a *Solanum lycopersicum* cv. M82 stem (scale bar = 1000 μm) in consecutive coils where different stages can be found (with youngest stages on top and oldest at the bottom). (B) Cross sections at the interface region between a *S. lycopersicum* cv. M82 stem and various *C. campestris* parasitizing stages (scale bar = 500 μm). Infection sites with several coils (H1 and H2) were divided into two or three subsamples (−1, −2, −3). Swelling (SWE) stages appeared as smooth to slightly bumped parasite stems (H1‐1, H2‐1, H4, H5, H7 and H8). Attaching (ATT) stages appeared as clearly bumped parasite stems with no visibly emerged haustoria (H1‐2, H2‐2 and H6). Penetrating (PEN) stages appeared with visible endophytic haustoria (H2‐3 and H3). (C) RT‐qPCR transcript accumulation transformed into *z*‐scores. Expression values were normalized against Cc028378.t1 and Cc002986.t1. Hierarchical clustering was based on Euclidean distance. A *z*‐score value is positive (negative) if the transcript accumulation in a sample is larger (smaller) than the mean row accumulation. niS, non‐infective stem

After normalization of transcript abundance, the selected markers mostly showed a consistent expression pattern in the parallel host‐free experiment compared with the RPKM values observed in the sequenced samples (Figures 3A,B and S4). Altogether, the marker triples allowed for a proper clustering of the parallel host‐free biological replicates (Figure 3C). Finally, for a proof of principle, 11 infection sites were randomly selected on *S. lycopersicum* M82, which is tolerant to *C. campestris*, and cross‐sectioned (Figure 4A,B). Visual inspection of the interface region classified those sites into 6 SWE, 3 ATT and 2 PEN. Three sections in distinct non‐infective stems, which were not in contact with the host nor exhibited any signs of development of infectious structures, were then included. Importantly, the transcript accumulation profiles of the selected markers remained in line with the morphological characteristics of the samples. Hierarchical clustering based on marker expression further classified the 11 infection sites into stages consistent with our visual observations of the interface region (Figure 4C), highlighting the suitability of the markers also in a host‐induced system.

## 4 DISCUSSION

One major breakthrough in functional genomic studies in dodders was the almost simultaneous release of two reference genomes (Sun et al., 2018; Vogel et al., 2018). Here, we built on this opportunity to generate a comprehensive and continuous host‐free transcriptome in *C. campestris* using RNA sequencing and explore the dynamic changes in gene expression. Unlike previous transcriptomic studies (Kaga et al., 2020; Olsen et al., 2016; Ranjan et al., 2014), we went beyond the simple distinction between young and mature haustoria, and defined three successive and macroscopically distinctive stages of development that can be induced without a host present: swelling, attaching and penetrating. The rationale behind the use of a host‐free induction system as used in this study, is that it greatly facilitated the sampling of morphologically and transcriptionally uniform samples with high reproducibility. The representation of mature haustoria that are in a feeding and interacting modus with the host is perhaps more limited, but with the refinement of the host‐free support system for *Cuscuta* that enables the parasite to feed on artificial surfaces (Bernal‐Galeano & Westwood, 2021), the reduction of the interaction system to just one biological partner is likely to become a standard in future studies on the biology of *Cuscuta*.

Multiple comparisons of stages indicated more DEGs in SWE than any other stage, supporting the involvement of numerous regulatory processes in the earliest step of haustoriogenesis. This is consistent with the profound morphological changes that are observed during the transition from niS to SWE. Haustorium inducing factors are known to trigger signal transduction cascades, mediating cell expansion and division in the cortical layers of the stem and resulting in the swelling at the initiation sites (Kokla & Melnyk, 2018). Here, protein‐ and hormone‐related categories were highlighted in SWE. That such pathways were activated indicates that the parasite perceived the external stimuli to which it was exposed and responded to them by inducing haustoriogenesis. Protein phosphorylation plays a major role in signal transduction. In plants, protein phosphorylation has been implicated in responses to many signals, including light, thanks to a large array of different protein kinases (Stone & Walker, 1995). The current data suggest a large contribution of leucine‐rich repeat (LRR) receptor‐like kinases, which are known to be involved in growth and development (Liu et al., 2017). Similarly, phytohormones are key components in the development of infective structures in parasitic plants (Kokla & Melnyk, 2018). These include auxin that, in the earlier steps, is known to accumulate at initiation sites and trigger the expression of numerous genes involved in haustorium formation. Among those genes are expansins that contribute to cell wall loosening and cell expansion. In *C. campestris*, auxin‐related biosynthetic enzymes and transporters (among others), as well as 11 of the 22 alpha‐class expansins that are part of the current transcriptome were upregulated in SWE. Further functional categories that were enriched in SWE, including cell cycle, cytoskeleton and cell wall, likely coordinate meristem development and organogenesis.

Beyond this initial SWE stage, the more subtle changes in gene expression may indicate that once the molecular cascade of events toward haustorial development has been started, only small adjustments are necessary to advance the process despite the more dramatic alterations in morphology. This appears to corroborate the intriguing observation that the FR induction can be reversed by red light only in the earliest stages of haustoriogenesis (Tada et al., 1996). The validity of ATT as a discernable stage, although relatively transient in our host‐free system and with remarkably few indicative DEGs, is sustained by the sequencing data. Functional categories related to pectin modification and degradation, representing genes that could promote host surface attachment, were enriched at this stage, consistent with the fact that the parasite is getting prepared for invasion (Yoshida et al., 2016). Because a host is lacking, there is no further incentive for the haustoria to differentiate beyond the penetrating stage, although feeding‐hyphae‐like structures can sometimes be observed when the haustorium is prevented from drying out (data not shown). Recent observations that a fluorescent dye was taken up with amazing speed by the haustoria and the surrounding adhesive ring (Lachner et al., 2020), support the notion that some form of uptake competence may already exist at this point. More investigations are needed in the future to explore this possibility. Haustoria that are induced in the absence of a host do not contain xylem bridges that are required to form vascular connections (Kaga et al., 2020; Yoshida et al., 2016). It should nevertheless be noted that among the hormone‐related functional categories that were enriched, TDIF peptide receptors (TDR) can be found, which are involved in cellular differentiation into tracheary elements (Morita et al., 2016). This could indicate that the parasite has already developed the competence to differentiate these structures that are crucial for its survival at this point and is awaiting the necessary cues from a host to proceed.

The analysis of expression patterns in the different stages revealed both constitutively expressed genes and genes with dramatic shifts in their expression. Further analyses were carried out to identify genes that can facilitate in vivo investigations of haustoriogenesis. Among common methods for gene expression analysis, RT‐qPCR has emerged as a fast, reliable and easy to use technique to measure transcript abundance in response to developmental variations and experimental conditions (Pabinger et al., 2014). Here, a new set of reference genes for RT‐qPCR was identified along with stage‐specific markers and validated in both a host‐free and host‐induced system. Some of the markers had no known function based on MapMan4 predictions, while the function of others reflects their involvement in haustoriogenesis and/or host invasion. For instance, Cc008389.t1 (PEN) is an alpha‐class cell wall expansin which (as mentioned earlier) is expected to play a role in the regulation of cell wall expansion, while Cc004177.t1 (PEN) is a berberine bridge enzyme‐like protein that possibly inactivates oligogalacturonides released upon pectin alteration and prevents them from triggering either the parasite or the host immunity (Benedetti et al., 2018). We considered three markers as a strict minimum for the molecular characterization of a sample and its assignment to one of the stages. This number can be seen as a balance between cost effectiveness and accuracy. It should be noted that the markers, although highly correlated with one of the development stages, often show weaker expression in others. It is therefore important that the entire set of markers is tested on a sample for its proper characterization. Also, infection sites can vary in length and a gradient in development stages among the haustoria can be observed. As a result, longer sites consisting of several coils must be split so that the less extensive the sites or the less haustoria per site or sample, the better for the marker pattern interpretation.

## AUTHOR CONTRIBUTIONS

Kirsten Krause and Julien Bruckmüller conceived and planned the research. Thomas Bawin and Kirsten Krause performed the experiments. Stian Olsen did the histological analyses. Thomas Bawin and Julien Bruckmüller performed the bioinformatics analyses. Thomas Bawin, Julien Bruckmüller, Stian Olsen, and Kirsten Krause interpreted the data. Thomas Bawin and Kirsten Krause drafted the first manuscript version. All authors contributed to finalizing the manuscript. All authors read and approved the final manuscript.

## Supporting information


**Figure S1**. Hierarchical clustering of sequenced biological replicates.
**Figure S2**. Cluster dendrogram of DEGs in host‐free haustorium development.
**Figure S3**. Pairwise variation V from geNorm.
**Figure S4**. Average transcript abundance of selected markers in parallel validation samples.
**Table S1**. Filtering and mapping statistics.
**Table S2**. Primer sequence pairs for the selected references with their amplicon sizes.
**Table S3**. Primer sequence pairs for the selected markers with their amplicon sizes.

**Appendix S1**. RNA sequencing dataset.

**Appendix S2**. RT‐qPCR expression in host‐free validation samples.

**Appendix S3**. RT‐qPCR expression in host‐induced samples.

## Data Availability

The generated raw reads from this article can be found in the NCBI Sequence Read Archive (SRA) under the accession number PRJNA666991.

## References

[ppl13628-bib-0001] Altschul, S.F. , Gish, W. , Miller, W. , Myers, E.W. & Lipman, D.J. (1990) Basic local alignment search tool. Journal of Molecular Biology, 215, 403–410. 2231712 10.1016/S0022-2836(05)80360-2

[ppl13628-bib-0002] Andersen, C.L. , Jensen, J.L. & Ørntoft, T.F. (2004) Normalization of real‐time quantitative reverse transcription‐PCR data: a model‐based variance estimation approach to identify genes suited for normalization, applied to bladder and colon cancer data sets. Cancer Research, 64, 5245–5250. 15289330 10.1158/0008-5472.CAN-04-0496

[ppl13628-bib-0003] Benedetti, M. , Verrascina, I. , Pontiggia, D. , Locci, F. , Mattei, B. , De Lorenzo, G. et al. (2018) Four Arabidopsis berberine bridge enzyme‐like proteins are specific oxidases that inactivate the elicitor‐active oligogalacturonides. The Plant Journal, 94, 260–273. 29396998 10.1111/tpj.13852

[ppl13628-bib-0004] Benjamini, Y. & Hochberg, Y. (1995) Controlling the false discovery rate: a practical and powerful approach to multiple testing. Journal of the Royal Statistical Society, Series B, 57, 289–300.

[ppl13628-bib-0005] Bernal‐Galeano, V. & Westwood, J.H. (2021) An artificial host system enables the obligate parasitic plant *Cuscuta campestris* to grow and complete its life cycle in vitro. bioRxiv. 10.1101/2021.06.21.449293 PMC9157073 35294033

[ppl13628-bib-0006] Costea, M. , García, M.A. & Stefanović, S. (2015) A phylogenetically based Infrageneric classification of the parasitic plant genus *Cuscuta* (Dodders, Convolvulaceae). Systematic Botany, 40, 269–285.

[ppl13628-bib-0007] Galloway, A.F. , Knox, P. & Krause, K. (2020) Sticky mucilages and exudates of plants: putative microenvironmental design elements with biotechnological value. The New Phytologist, 225, 1461–1469. 31454421 10.1111/nph.16144

[ppl13628-bib-0008] García, M.A. , Costea, M. , Kuzmina, M. & Stefanović, S. (2014) Phylogeny, character evolution, and biogeography of *Cuscuta* (Dodders; Convolvulaceae) inferred from coding plastid and nuclear sequences. American Journal of Botany, 101, 670–690. 24688058 10.3732/ajb.1300449

[ppl13628-bib-0009] Haidar, M.A. (2003) Characterisation of the interaction between cryptochromes and phytochromes in blue light‐induced coiling and prehaustoria development of dodder (*Cuscuta campestris*) seedlings. The Annals of Applied Biology, 143, 57–62.

[ppl13628-bib-0010] Jhu, M.‐Y. , Ichihashi, Y. , Farhi, M. , Wong, C. & Sinha, N.R. (2021) Lateral organ boundaries domain 25 functions as a key regulator of haustorium development in dodders. Plant Physiology, 186, 2093–2110. 34618110 10.1093/plphys/kiab231 PMC8331169

[ppl13628-bib-0011] Johnsen, H.R. , Striberny, B. , Olsen, S. , Vidal‐Melgosa, S. , Fangel, J.U. , Willats, W.G.T. et al. (2015) Cell wall composition profiling of parasitic giant dodder (*Cuscuta reflexa*) and its hosts: a priori differences and induced changes. The New Phytologist, 207, 805–816. 25808919 10.1111/nph.13378

[ppl13628-bib-0012] Kaga, Y. , Yokoyama, R. , Sano, R. , Ohtani, M. , Demura, T. , Kuroha, T. et al. (2020) Interspecific signaling between the parasitic plant and the host plants regulate xylem vessel cell differentiation in haustoria of *Cuscuta campestris* . Frontiers in Plant Science, 11, 193. 32231674 10.3389/fpls.2020.00193 PMC7082356

[ppl13628-bib-0013] Kim, G. & Westwood, J.H. (2015) Macromolecule exchange in *Cuscuta*–host plant interactions. Current Opinion in Plant Biology, 26, 20–25. 26051214 10.1016/j.pbi.2015.05.012

[ppl13628-bib-0014] Kokla, A. & Melnyk, C.W. (2018) Developing a thief: haustoria formation in parasitic plants. Developmental Biology, 442, 53–59. 29935146 10.1016/j.ydbio.2018.06.013

[ppl13628-bib-0015] van der Kooij, T.A. , Krause, K. , Dörr, I. & Krupinska, K. (2000) Molecular, functional and ultrastructural characterisation of plastids from six species of the parasitic flowering plant genus *Cuscuta* . Planta, 210, 701–707. 10805440 10.1007/s004250050670

[ppl13628-bib-0016] Lachner, L.A. , Galstyan, L. & Krause, K. (2020) A highly efficient protocol for transforming *Cuscuta reflexa* based on artificially induced infection sites. Plant Direct, 4(8), e00254. 32789286 10.1002/pld3.254 PMC7417715

[ppl13628-bib-0017] Langfelder, P. & Horvath, S. (2008) WGCNA: an R package for weighted correlation network analysis. BMC Bioinformatics, 9, 559. 19114008 10.1186/1471-2105-9-559 PMC2631488

[ppl13628-bib-0018] Lee, K.B. (2007) Structure and development of the upper haustorium in the parasitic flowering plant *Cuscuta japonica* (Convolvulaceae). American Journal of Botany, 94, 737–745. 21636442 10.3732/ajb.94.5.737

[ppl13628-bib-0019] Lee, K.B. (2008) Anatomy and ultrastructure of epidermal cells in the haustorium of a parasitic flowering plant, Cuscuta japonica, during attachment to the host. Journal of Plant Biology, 51, 366–372.

[ppl13628-bib-0020] Liu, P.‐L. , Du, L. , Huang, Y. , Gao, S.‐M. & Yu, M. (2017) Origin and diversification of leucine‐rich repeat receptor‐like protein kinase (LRR‐RLK) genes in plants. BMC Evolutionary Biology, 17, 47. 28173747 10.1186/s12862-017-0891-5 PMC5296948

[ppl13628-bib-0021] Liu, N. , Shen, G. , Xu, Y. , Liu, H. , Zhang, J. , Li, S. et al. (2020) Extensive inter‐plant protein transfer between *Cuscuta* parasites and their host plants. Molecular Plant, 13, 573–585. 31812691 10.1016/j.molp.2019.12.002

[ppl13628-bib-0022] Morita, J. , Kato, K. , Nakane, T. , Kondo, Y. , Fukuda, H. , Nishimasu, H. et al. (2016) Crystal structure of the plant receptor‐like kinase TDR in complex with the TDIF peptide. Nature Communications, 7, 12383. 10.1038/ncomms12383 PMC4979064 27498761

[ppl13628-bib-0023] Narukawa, H. , Yokoyama, R. , Kuroha, T. & Nishitani, K. (2021) Host‐produced ethylene is required for marked cell expansion and endoreduplication in dodder search hyphae. Plant Physiology, 185, 491–502. 33721891 10.1093/plphys/kiaa010 PMC8133569

[ppl13628-bib-0024] Olsen, S. , Striberny, B. , Hollmann, J. , Schwacke, R. , Popper, Z. & Krause, K. (2016) Getting ready for host invasion: elevated expression and action of xyloglucan endotransglucosylases/hydrolases in developing haustoria of the holoparasitic angiosperm *Cuscuta* . Journal of Experimental Botany, 67, 695–708. 26561437 10.1093/jxb/erv482 PMC4737069

[ppl13628-bib-0025] Pabinger, S. , Rödiger, S. , Kriegner, A. , Vierlinger, K. & Weinhäusel, A. (2014) A survey of tools for the analysis of quantitative PCR (qPCR) data. Biomolecular Detection and Quantification, 1, 23–33. 27920994 10.1016/j.bdq.2014.08.002 PMC5129434

[ppl13628-bib-0026] Perkins, J.R. , Dawes, J.M. , McMahon, S.B. , Bennett, D.L.H. , Orengo, C. & Kohl, M. (2012) ReadqPCR and NormqPCR: R packages for the reading, quality checking and normalisation of RT‐qPCR quantification cycle (Cq) data. BMC Genomics, 13, 296. 22748112 10.1186/1471-2164-13-296 PMC3443438

[ppl13628-bib-0027] Pfaffl, M.W. , Tichopad, A. , Prgomet, C. & Neuvians, T.P. (2004) Determination of stable housekeeping genes, differentially regulated target genes and sample integrity: BestKeeper—excel‐based tool using pair‐wise correlations. Biotechnology Letters, 26, 509–515. 15127793 10.1023/b:bile.0000019559.84305.47

[ppl13628-bib-0028] Ranjan, A. , Ichihashi, Y. , Farhi, M. , Zumstein, K. , Townsley, B. , David‐Schwartz, R. et al. (2014) De novo assembly and characterization of the transcriptome of the parasitic weed dodder identifies genes associated with plant parasitism. Plant Physiology, 166, 1186–1199. 24399359 10.1104/pp.113.234864 PMC4226353

[ppl13628-bib-0029] Robinson, M.D. , McCarthy, D.J. & Smyth, G.K. (2010) edgeR: a Bioconductor package for differential expression analysis of digital gene expression data. Bioinformatics, 26, 139–140. 19910308 10.1093/bioinformatics/btp616 PMC2796818

[ppl13628-bib-0030] Schwacke, R. , Ponce‐Soto, G.Y. , Krause, K. , Bolger, A.M. , Arsova, B. , Hallab, A. et al. (2019) MapMan4: a refined protein classification and annotation framework applicable to multi‐omics data analysis. Molecular Plant, 12, 879–892. 30639314 10.1016/j.molp.2019.01.003

[ppl13628-bib-0031] Shimizu, K. & Aoki, K. (2019) Development of parasitic organs of a stem holoparasitic plant in genus *Cuscuta* . Frontiers in Plant Science, 10, 1435. 31781146 10.3389/fpls.2019.01435 PMC6861301

[ppl13628-bib-0032] Stone, J.M. & Walker, J.C. (1995) Plant protein kinase families and signal transduction. Plant Physiology, 108, 451–457. 7610156 10.1104/pp.108.2.451 PMC157363

[ppl13628-bib-0033] Sun, G. , Xu, Y. , Liu, H. , Sun, T. , Zhang, J. , Hettenhausen, C. et al. (2018) Large‐scale gene losses underlie the genome evolution of parasitic plant *Cuscuta australis* . Nature Communications, 9, 2683. 10.1038/s41467-018-04721-8 PMC6041341 29992948

[ppl13628-bib-0034] Švubová, R. & Blehová, A. (2013) Stable transformation and actin visualization in callus cultures of dodder (*Cuscuta europaea*). Biologia (Bratisl), 68, 68–640.

[ppl13628-bib-0035] Tada, Y. , Sugai, M. & Furuhashi, K. (1996) Haustoria of *Cuscuta japonica*, a Holoparasitic flowering plant, are induced by the cooperative effects of far‐red light and tactile stimuli. Plant & Cell Physiology, 37, 1049–1053.

[ppl13628-bib-0036] Untergasser, A. , Cutcutache, I. , Koressaar, T. , Ye, J. , Faircloth, B.C. , Remm, M. et al. (2012) Primer3—new capabilities and interfaces. Nucleic Acids Research, 40, e115. 22730293 10.1093/nar/gks596 PMC3424584

[ppl13628-bib-0037] Vandesompele, J. , De Preter, K. , Pattyn, F. , Poppe, B. , Van Roy, N. , De Paepe, A. et al. (2002) Accurate normalization of real‐time quantitative RT‐PCR data by geometric averaging of multiple internal control genes. Genome Biology, 3, RESEARCH0034. 12184808 10.1186/gb-2002-3-7-research0034 PMC126239

[ppl13628-bib-0038] Vaughn, K.C. (2002) Attachment of the parasitic weed dodder to the host. Protoplasma, 219, 227–237. 12099223 10.1007/s007090200024

[ppl13628-bib-0039] Vaughn, K.C. (2003) Dodder hyphae invade the host: a structural and immunocytochemical characterization. Protoplasma, 220, 189–200. 12664283 10.1007/s00709-002-0038-3

[ppl13628-bib-0040] Vaughn, K.C. (2006) Conversion of the searching hyphae of dodder into xylic and phloic hyphae: a cytochemical and immunocytochemical investigation. International Journal of Plant Sciences, 167, 1099–1114.

[ppl13628-bib-0041] Vogel, A. , Schwacke, R. , Denton, A.K. , Usadel, B. , Hollmann, J. , Fischer, K. et al. (2018) Footprints of parasitism in the genome of the parasitic flowering plant *Cuscuta campestris* . Nature Communications, 9, 2515. 10.1038/s41467-018-04344-z PMC6023873 29955043

[ppl13628-bib-0042] Vurro, M. , Pérez‐de‐Luque, A. & Eizenberg, H. (2017) Parasitic weeds. Weed research. Chichester, UK: John Wiley & Sons, pp. 313–353.

[ppl13628-bib-0043] Yoshida, S. , Cui, S. , Ichihashi, Y. & Shirasu, K. (2016) The haustorium, a specialized invasive organ in parasitic plants. Annual Review of Plant Biology, 67, 643–667. 10.1146/annurev-arplant-043015-111702 27128469

