# Supplementary material for: A host‐free transcriptome for haustoriogenesis in Cuscuta campestris: Signature gene expression identifies markers of successive development stages
Source: Physiol Plant. 2022 Mar 6;174(2):e13628. doi: 10.1111/ppl.13628 (PMC9313786; doi:10.1111/ppl.13628)
Supplement: Supplementary file 1 — Figure S1. Hierarchical clustering of sequenced biological replicates. Figure S2. Cluster dendrogram of DEGs in host‐free haustorium development. Figure S3. Pairwise variation V from geNorm. Figure S4. Average transcript abundance of selected markers in parallel validation samples. Table S1. Filtering and mapping statistics. Table S2. Primer sequence pairs for the selected references with their amplicon sizes. Table S3. Primer sequence pairs for the selected markers with their amplicon sizes. [file PPL-174-0-s002.docx]

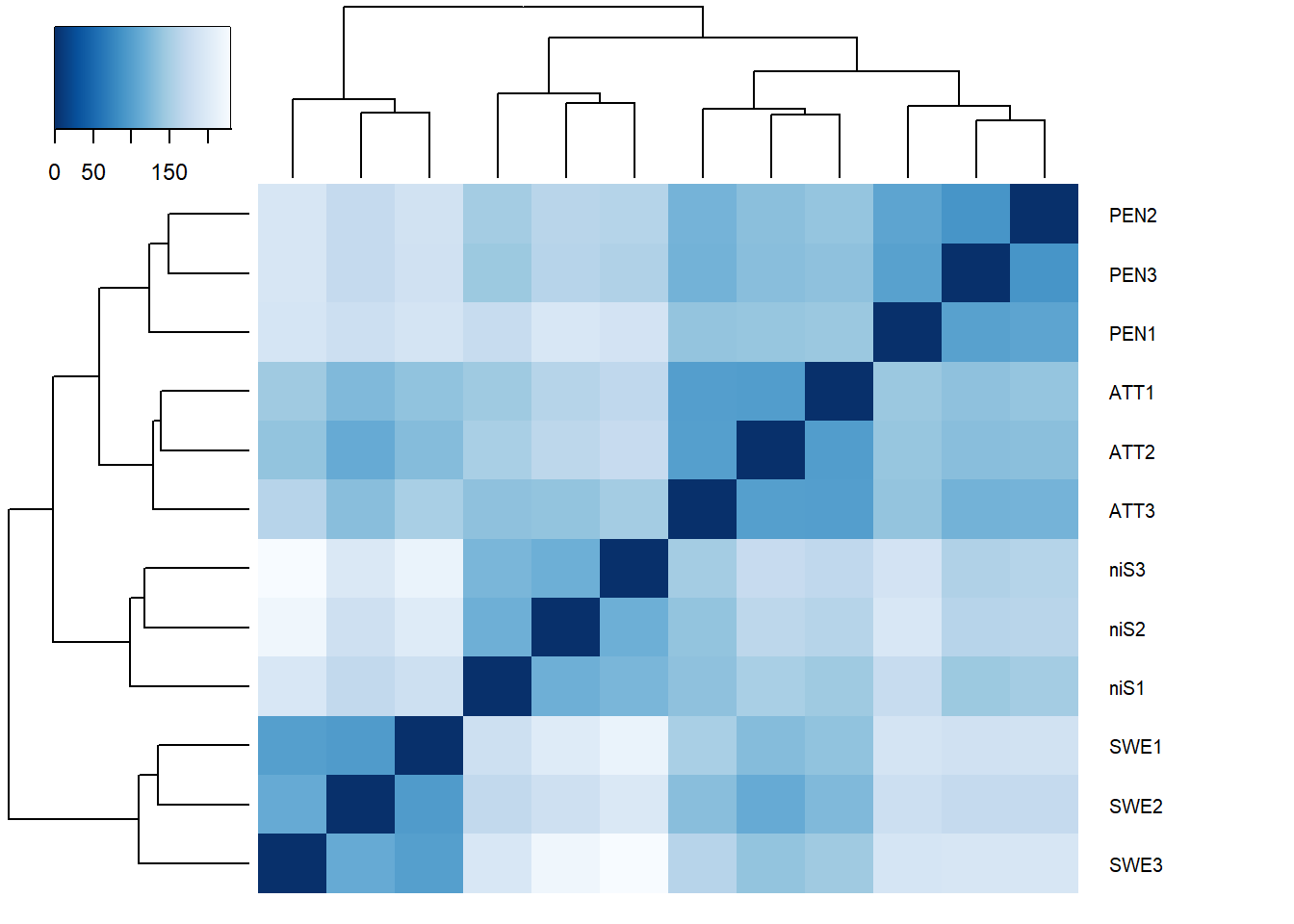


**Fig. S1. Hierarchical clustering of sequenced biological replicates.** Calculations are based on Euclidean distance between log_2_(counts-per-million + pseudocount) values. Note the map is symmetrical, for the samples are plotted against each other. The darker the color is, the closer the samples are. niS = non-infective stem; SWE = swelling stage; ATT = attaching stage; PEN = penetrating stage.


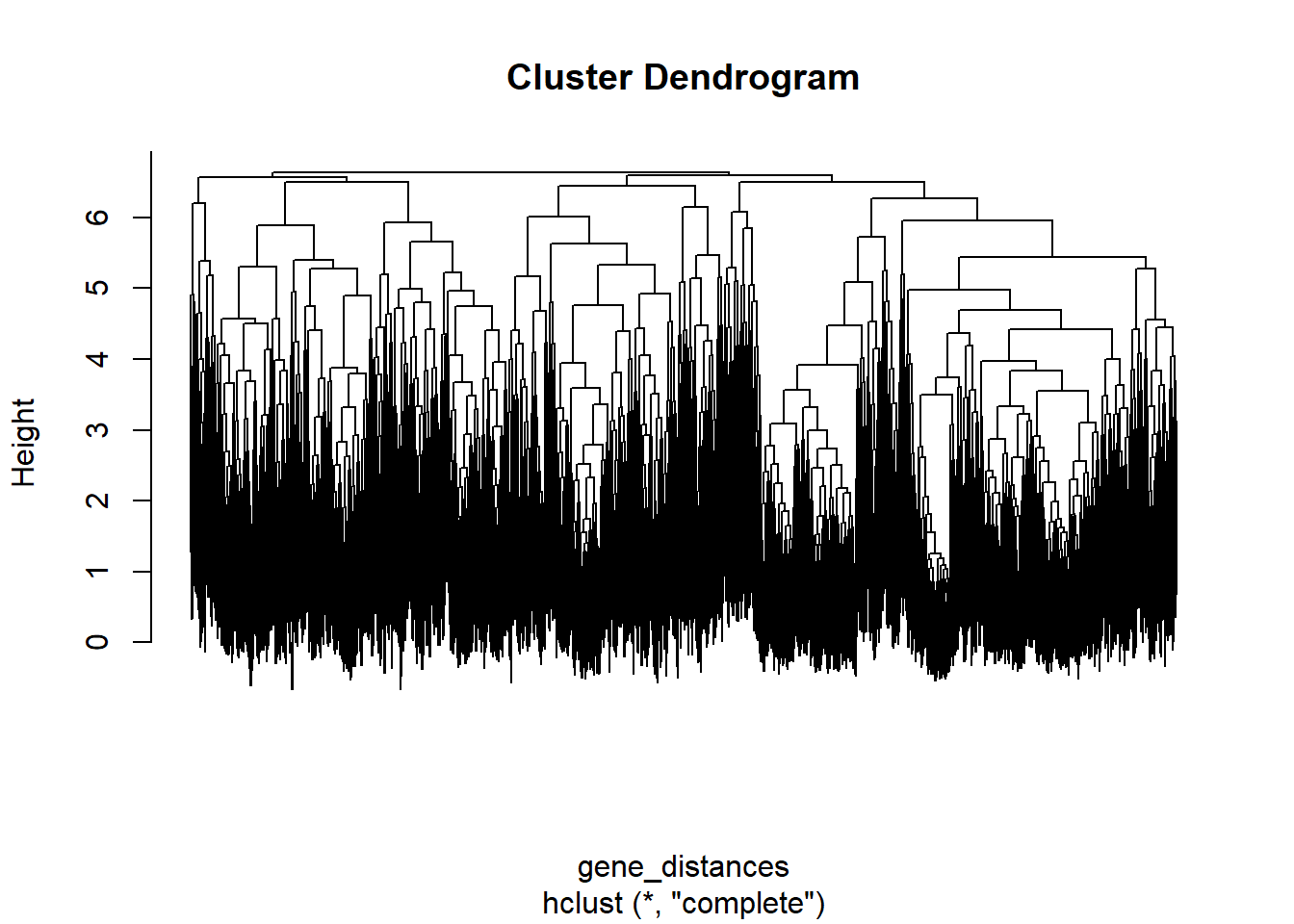


**Fig. S2. Cluster dendrogram of DEGs in host-free haustorium development.** Clustering was performed by applying the complete-linkage method to a Euclidean distance matrix (based on expression values). Relates to Fig. 2A.


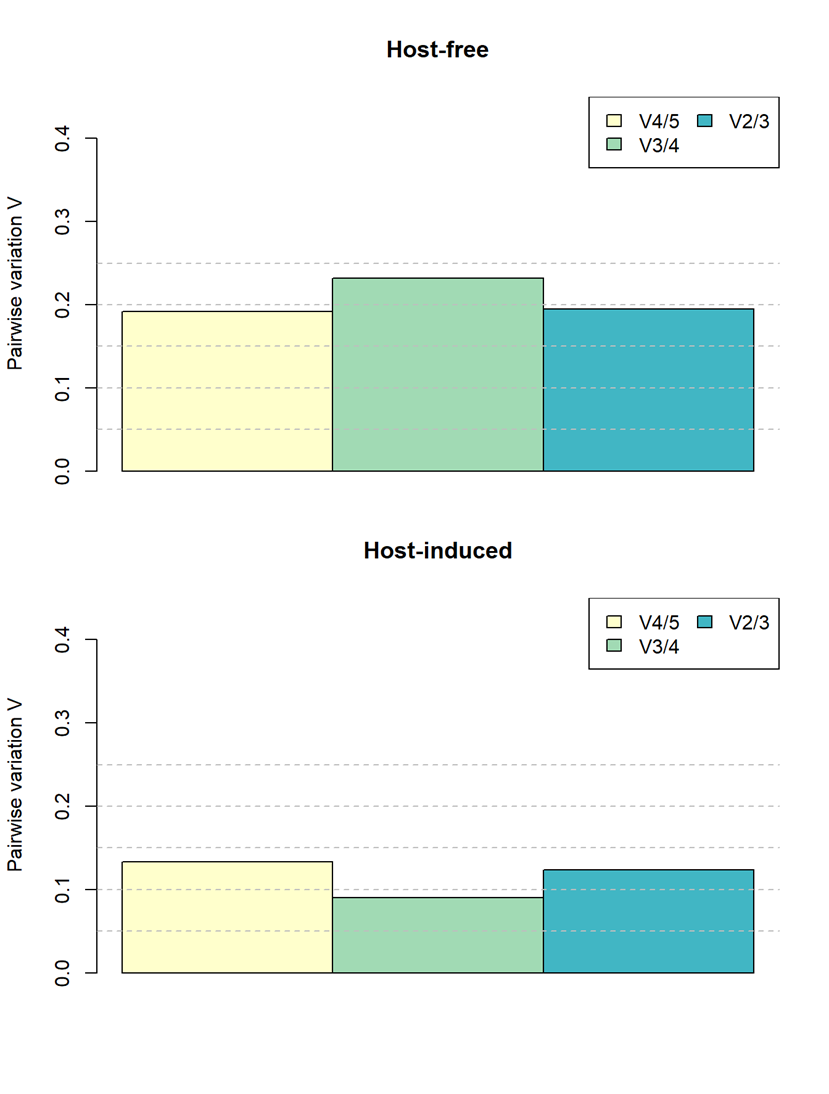


**Fig. S3. Pairwise variation V from geNorm.** Briefly, the expression stability M was used to rank the selected housekeepers and the pairwise variation V was used to determine the minimum number to be included for the reliable normalization of a dataset. Vn/n+1 refers to the pairwise variation between the normalization factors NFn and NFn+1. The lower the Vn/n+1 value, the better the normalization of a dataset can be expected. For details, see Vandesompele et al. (2002).


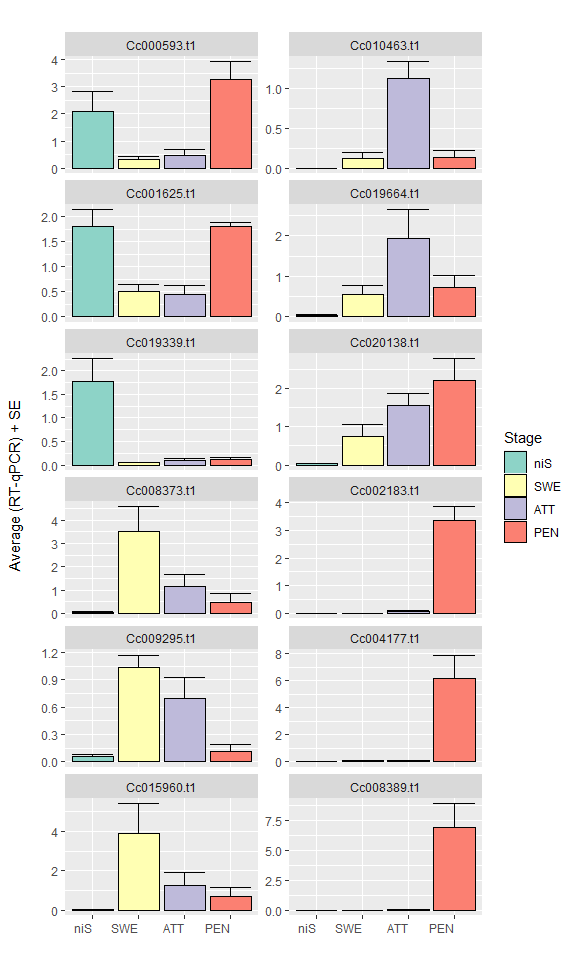


**Fig. S4. Average transcript abundance of selected markers in parallel validation samples.** Expression values (as measured by RT-qPCR) were normalized against Cc028808.t1 and Cc006757.t1. Error bars indicate standard error of the mean (three biological replicates). niS = non-infective stem; SWE = swelling stage; ATT = attaching stage; PEN = penetrating stage.

**Table S1. Filtering and mapping statistics.**

| **Sample** | **Total Bases** | **Raw Reads** | **GC (%)** | **AT (%)** | **Q20 (%)** | **Q30 (%)** | **Trimmed** | **Trimmed (%)** | **Mapped**  **(pairs)** | **Mapped**  **(broken pairs)** | **Total mapped** | **Total Mapped (%)** |
| --- | --- | --- | --- | --- | --- | --- | --- | --- | --- | --- | --- | --- |
| niS1 | 3 095 188 026 | 30 645 426 | 46.96 | 53.04 | 97.70 | 96.27 | 30 643 802 | 99.995 | 18 024 480 | 3 498 850 | 21 523 330 | 70.24 |
| niS2 | 3 119 651 438 | 30 887 638 | 46.78 | 53.22 | 97.91 | 96.52 | 30 885 850 | 99.994 | 16 501 778 | 4 566 934 | 21 068 712 | 68.21 |
| niS3 | 3 176 907 328 | 31 454 528 | 47.14 | 52.86 | 97.72 | 96.29 | 31 452 844 | 99.995 | 16 586 222 | 5 011 883 | 21 598 105 | 68.67 |
| SWE1 | 3 187 891 078 | 31 563 278 | 47.34 | 52.66 | 97.90 | 96.50 | 31 561 590 | 99.995 | 19 165 096 | 4 050 356 | 23 215 452 | 73.56 |
| SWE2 | 3 194 538 292 | 31 629 092 | 46.96 | 53.04 | 97.93 | 96.54 | 31 627 460 | 99.995 | 17 287 342 | 5 353 166 | 22 640 508 | 71.58 |
| SWE3 | 3 181 221 846 | 31 497 246 | 46.67 | 53.33 | 97.87 | 96.49 | 31 495 334 | 99.994 | 17 820 286 | 4 552 980 | 22 373 266 | 71.04 |
| ATT1 | 3 168 909 744 | 31 375 344 | 47.30 | 52.70 | 97.83 | 96.40 | 31 373 812 | 99.995 | 17 423 660 | 5 281 410 | 22 705 070 | 72.37 |
| ATT2 | 3 175 595 136 | 31 441 536 | 47.14 | 52.86 | 97.86 | 96.45 | 31 439 922 | 99.995 | 17 629 102 | 4 893 330 | 22 522 432 | 71.64 |
| ATT3 | 3 094 174 592 | 30 635 392 | 47.15 | 52.85 | 97.89 | 96.50 | 30 633 726 | 99.995 | 17 494 228 | 4 459 640 | 21 953 868 | 71.67 |
| PEN1 | 3 121 864 146 | 30 909 546 | 47.51 | 52.49 | 97.94 | 96.54 | 30 908 096 | 99.995 | 17 234 600 | 5 149 323 | 22 383 923 | 72.42 |
| PEN2 | 3 204 659 300 | 31 729 300 | 47.28 | 52.72 | 97.89 | 96.51 | 31 727 534 | 99.994 | 18 128 132 | 4 816 872 | 22 945 004 | 72.32 |
| PEN3 | 3 130 530 552 | 30 995 352 | 47.19 | 52.81 | 97.82 | 96.38 | 30 993 546 | 99.994 | 18 453 904 | 3 510 120 | 21 964 024 | 70.87 |

**Table S2. Primer sequence pairs for the selected references with their amplicon sizes.**

| **Accession** | **Forward (5'-3')** | **Reverse (5'-3')** | **Amplicon (pb)** |
| --- | --- | --- | --- |
| Cc002986.t1 | CCCACAGCTCCTCCATTTGT | CACCGCACGCCAAACTTTAA | 114 |
| Cc006757.t1 | AGAGGTAGAGCTTGTTGCCA | TGATGATGGTGCCAAAGGGG | 88 |
| Cc028378.t1 | TAGGCCGGAGCTTGTGAAAG | GATTGGTGCACAAGCCCTTG | 197 |
| Cc028808.t1 | TGTGCAAGCAACCTTTCACC | CTTCCATGGGTGCCTAAGCT | 91 |
| Cc036327.t1 | ACGGCTGCAACAAGAGGATT | AGGCATGGGAATGGAAACGA | 182 |

**Table S3. Primer sequence pairs for the selected markers with their amplicon sizes.**

| **Accession** | **Forward (5'-3')** | **Reverse (5'-3')** | **Amplicon (pb)** |
| --- | --- | --- | --- |
| Cc000593.t1 | AAACACCGGCATCTTCGACT | GAACACTCTTCTGGCGGGAA | 133 |
| Cc001625.t1 | GCGAAGTGCTACCCTCAGTT | CCCTTGATAGGTGATCCCGC | 160 |
| Cc019339.t1 | GACGTGCTGGTGCCATAGAT | GCAATTAGCTCCTCCCCCTC | 133 |
| Cc008373.t1 | AGGATTGATGACGGGGGCTA | TGGTTCGCTTCAAAGGAGCT | 151 |
| Cc009295.t1 | CCGCTCGCTTCCTCCATTAT | CCAAAACCGGCTTCCAAGTG | 79 |
| Cc015960.t1 | CAGCTATAGAACAGCACCCGT | TGTGAAGCAACAGCCCAGAA | 95 |
| Cc010463.t1 | GCTCTCCCTTCACTCCCAAC | GTGGAGAGATTTTGAGCGACAG | 161 |
| Cc019664.t1 | TGGGGAAGAAATCGACGTGT | ACCCAACCGACTCGATCTTC | 161 |
| Cc020138.t1 | TTTCCCAATCAAGTCCCCGG | AGTGACCATCTTGCCCATGC | 121 |
| Cc002183.t1 | TAACCCTTCCACGCACTGTC | TCACCCACAATAGCGCAGTT | 178 |
| Cc004177.t1 | TCTGATGGGAATTCCGTTCGC | GTCTGGCTCGTGAGATCGAC | 75 |
| Cc008389.t1 | GCCTCAAATGTCAGTTGCGA | TCACGACAATAGAGCCTCCC | 250 |

**REFERENCES**

Vandesompele J, De Preter K, Pattyn F, Poppe B, Van Roy N, De Paepe A, Speleman F (2002) Accurate normalization of real-time quantitative RT-PCR data by geometric averaging of multiple internal control genes. Genome Biol 3: RESEARCH0034
